# Accuracy of curability expectations in patients with gastrointestinal cancers

**DOI:** 10.1002/cam4.4947

**Published:** 2022-06-15

**Authors:** Rajiv Agarwal, Paul Shin, Andrea Knezevic, Judith E. Nelson, Danielle R. Romano, Camila Bernal, Anjali V. Desai, Andrew S. Epstein

**Affiliations:** ^1^ Department of Medicine Vanderbilt University Medical Center Nashville Tennessee USA; ^2^ Memorial Sloan Kettering Cancer Center New York New York USA; ^3^ Weill Cornell Medical College New York New York USA

**Keywords:** advance care planning, communication, gastrointestinal cancers, informed consent, prognostic awareness

## Abstract

**Background:**

Assessment of illness and treatment understanding among cancer patients has largely focused on those with advanced disease. Less is known about patient expectations at earlier stages of cancer and potential modifiers of accurate understanding.

**Methods:**

We assessed accuracy of cure expectations in patients across all stages with gastrointestinal (GI) cancers. Accuracy was determined by independent reviews of patient health records by oncologists on the investigative team. Impact on cure accuracy of selected clinical variables and health‐information preferences was analyzed.

**Results:**

Hundred and thirty five patients were included for analysis, with 100% interrater agreement for accuracy between oncologist reviewers. Sixety five patients (48%) had accurate cure expectations from their cancer treatment. Accuracy was lower in Stage IV versus Stage I–III disease (35% vs. 63%, *p* < 0.01), lower in unresectable versus resectable disease (35% vs. 67%, *p* < 0.01), and higher in patients with early‐stage disease who received adjuvant chemotherapy versus those who did not (78% vs. 53%, *p* = 0.04). Accuracy did not differ by health‐information preferences and remained stable over time. Of 63 patients who died, baseline accuracy differed by location of death (*p* = 0.03), with greater accuracy in those who died with home hospice (56%). Accuracy was lower in those who were hospitalized in the last 30 days of life versus those who were not (25% vs. 59%, *p* = 0.01).

**Conclusions:**

Inaccurate cure expectations are prevalent across all stages of GI cancers, but particularly among those with metastatic or unresectable disease. High‐quality, iterative communication strategies may facilitate patient illness and treatment understanding throughout the disease course.

## Graphical abstract

Inaccurate cure expectations are prevalent among patients with all stages of gastrointestinal cancers, but particularly among those with metastatic or unresectable disease, and among early‐stage patients who did not receive adjuvant chemotherapy. High‐quality, iterative communication by clinicians may help patients better understand the course of their disease and intent of their cancer treatments.

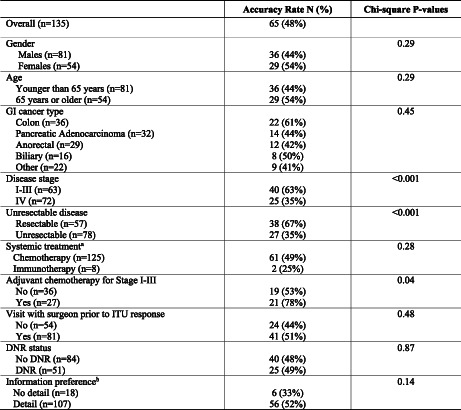

## 1 INTRODUCTION

Understanding the intent of cancer treatment is imperative for patients' informed consent and medical decision‐making. Patients therefore, deserve to receive prognostic and treatment‐related information, when desired, from their oncology teams in a clear and personalized manner.^1^, ^2^ Prognostic awareness and acknowledgment of the uncertainty about life expectancy can help patients plan for their future and make decisions according to their personal values and goals.^3^, ^4^ Though receipt of prognostic information may be distressing,^5^, ^6^, ^7^, ^8^ skillful communication by clinicians can support patients to integrate this information while preserving hope and psychological wellbeing.^9^, ^10^, ^11^

Despite the benefits of serious‐illness and prognostic discussions to increase awareness,^12^, ^13^, ^14^ patients may still have misunderstandings about their illness severity and the intent of cancer treatment. In a landmark study by Weeks et al, most patients with newly‐diagnosed metastatic lung or colorectal cancers expected to be cured from chemotherapy.^15^ Unrealistic expectations regarding curability may result from ineffective communication, the choice to not believe that one's disease is incurable, and/or the use of optimism as a coping strategy.^16^ Nevertheless, oncology care teams need to conduct high‐quality, empathic, and iterative conversations about the realistic goals of cancer treatment, in order for patients and families to feel supported and informed throughout the disease course.

Historically, research has focused on evaluating illness and treatment understanding in patients with metastatic or advanced cancer. Evidence about expectations of patients with earlier stage cancer is scant. In addition, little is known about an individual patient's evolution of their expectations over time and potential modifiers of, and associations with, accurate understanding. Moreover, the prior research predated the groundbreaking progress of molecularly‐driven and immune‐based cancer therapies that have improved survival outcomes, and was limited to a one‐time survey assessment for patients who received chemotherapy.^15^ A contemporary evaluation of illness and treatment understanding in our current era of cancer therapeutics and in the context of real‐world clinical practice is needed. We therefore, analyzed the accuracy of cure expectations in patients across all stages of gastrointestinal (GI) cancers and characterized accuracy in relation to defined clinical variables and health‐information preferences. We further examined whether expectations changed over time and if accuracy varied by end‐of‐life care outcomes.

## 2 METHODS

This prospective cohort study was conducted at Memorial Sloan Kettering Cancer Center (MSK) as a pre‐planned component of a clinical initiative to normalize, systematize, and improve the quality of “primary” (non‐specialist) palliative care by oncology teams supported by palliative care specialists.^17^, ^18^, ^19^ The study cohort consisted of patients with newly‐diagnosed GI cancers, regardless of disease stage, receiving care from medical oncology clinics from July 2017 to December 2020. For this analysis, study participants were followed until death or August 31, 2021, whichever occurred sooner. There were no exclusion criteria. MSK's Institutional Review Board waived informed consent based on minimal risk to participants.

As previously described, the larger clinical initiative comprised a structured, sequenced, and iterative series of assessments by outpatient oncology nurses to elicit patients' core health‐related values, symptom burden, and understanding of their illness and intent of cancer treatment.^17^, ^18^ To assess illness and treatment understanding (ITU), patients were asked three questions derived from a validated questionnaire used in a large national observational study of outcomes including perceptions of care among patients with metastatic colorectal and lung cancer.^15^ Specifically, patients used a 4‐point Likert scale to rate the likelihood (“*not at all likely*,” “*a little likely*,” “*somewhat likely*,” “*very likely*”) that their treatment would: (1) Cure their cancer; (2) prolong life; and (3) help with problems caused by the cancer.^15^ Along with these items, all patients were asked if they preferred to receive information about their health in detail or as an overview without much detail,^17^ and a subset of study patients were asked to assess the quality of communication provided by their treating oncology care teams, using a validated questionnaire (e.g., “how often does your care team… listen carefully to you, explain things in a way you could understand, give you as much information as you wanted about your cancer treatments?” and “how good is your care team at… involving you in treatment discussions about your care, asking you about important things in life, discussing your values/goals?”).^20^, ^21^, ^22^ Questionnaires were administered in person in clinic, initially at the second follow‐up clinic visit and then quarterly to evaluate changes in patient responses over time.

The primary objectives of this analysis were to assess the accuracy of patients' reported expectations for the likelihood of cure× from their cancer treatment at baseline, and to determine if accuracy rates varied by clinical variables and health information preferences. Two gastrointestinal medical oncologists (R.A. and A.S.E.) and one surgical oncology fellow (P.S.) independently assessed accuracy of patients' responses. Accuracy was determined by first review of patient data, including: GI cancer type, disease stage, recurrent or newly diagnosed disease, resectability, and receipt of neoadjuvant versus adjuvant versus metastatic treatment. The oncology reviewers then used clinical practice standards to classify the intent of cancer treatment for each patient's clinical scenario as either curative or non‐curative.

Patient responses were classified as accurate if their cure expectations were consistent with the intent of treatment determined by the oncology reviewers, and patient responses were classified as inaccurate if their cure expectations were unrealistic compared with reviewers' projections. For example, a patient with Stage IIIA colon cancer receiving adjuvant chemotherapy who reported that their cancer treatment was “*somewhat likely*” to be curative was deemed to have answered accurately; a patient with Stage IV pancreas cancer receiving chemotherapy who reported that their cancer treatment was “*very likely*” to be curative was deemed have answered inaccurately; a patient with Stage IV gastric cancer receiving combined chemotherapy and immunotherapy who reported that their cancer treatment was “*not at all likely*” to be curative was deemed to have answered accurately.

Independent assessments of the accuracy of patients' responses were then compared to each other to determine the degree of interrater agreement. When assessments of accuracy were not in agreement, these clinical cases were jointly reviewed to arrive at a consensus. Patients' responses were subsequently coded as accurate or inaccurate.

For study participants who completed the ITU questionnaire more than once (i.e., at baseline and a subsequent quarterly interval), change in accuracy was evaluated by comparison of baseline and the last completed questionnaire response. For those who died within the study period, electronic health records were reviewed to assess the following outcomes: location of death, hospice enrollment, receipt of cancer treatment within the last 30 days of life, and hospitalizations within the last 30 days of life. Accuracy of cure expectations was then evaluated relative to each end‐of‐life outcome.

### 2.1 Statistical analysis

Participant responses to the ITU, information preferences, and quality of communication questionnaires were summarized descriptively. Rate of cure expectation accuracy was estimated among all patients at baseline. Baseline cure accuracy was compared between groups with the Chi‐square test, including by demographic and clinical variables, for patient‐reported information preferences (for information in detail vs. no detail), and for end‐of‐life outcomes in those who died. Longitudinal data on curability expectations were used to identify changes in accuracy over time (either more or less accurate compared to reviewer classification) and reported descriptively. All tests were evaluated for statistical significance at alpha level 0.05. Statistical analysis was performed using SAS version 9.4.

## 3 RESULTS

### 3.1 Study participants

A total of 135 patients with GI cancers were available for analysis. Demographic and disease characteristics are described in Table 1. The median age of the study cohort was 61 years, with more men (60%) than women. Most patients identified as white (75%) and Non‐Hispanic/Non‐Latinx (94%). Most patients were married (66%). There was diversity among patients who identified with a religion (73%). Slightly more than half of the patients in the cohort had Stage IV disease and slightly less than half had Stage I–III disease. All patients completed the ITU questionnaire at baseline. The information preferences and quality of communication questionnaires were completed at baseline by 125 (93%) and 60 (44%) patients, respectively. In addition, 73 (54%) patients completed the ITU questionnaire on at least one follow‐up visit (median 3 questionnaires completed, range 2–7) and were included for longitudinal analysis.

**TABLE 1:** Patient demographics

Baseline characteristics	Patients (*N* = 135)
Age (median age in years, range)	61 (25–87)
Gender	
Male	81 (60%)
Female	54 (40%)
GI malignancy type	
Colon	36 (27%)
Pancreas adenocarcinoma	32 (24%)
Anorectal	29 (21%)
Biliary (gallbladder or cholangiocarcinoma)	16 (12%)
Other^a^	22 (16%)
Stage	
I	4 (3%)
II	13 (10%)
III	46 (34%)
IV	72 (53%)
Unresectable disease	
Resectable	57 (42%)
Unresectable	78 (58%)
Race	
White	101 (75%)
Asian	16 (12%)
Black/African American	11 (8%)
Other	6 (4%)
Native American	1 (1%)
Ethnicity	
Non‐Hispanic/Non‐Latinx	127 (94%)
Hispanic/Non‐Latinx	8 (6%)
Marital status^b^	
Married	89 (66%)
Single	23 (17%)
Divorced	10 (7%)
Widowed	8 (6%)
Life partner	3 (2%)
Separated	1 (1%)
Religion	
Roman catholic	45 (33%)
None	36 (27%)
Other Christian	26 (19%)
Jewish	19 (14%)
Unknown	4 (3%)
Muslim	2 (1.5%)
Hindu	2 (1.5%)
Buddhist	1 (1%)

Characteristics of the overall study population. Data presented as *N* (%) unless otherwise specified.

Abbreviations: GI, gastrointestinal.

^a^ Other GI malignancy types include: gastroesophageal (10), neuroendocrine (8), ampullary and small bowel (3), and adrenocortical carcinoma (1).

^b^ Unknown for 1 patient.

### 3.2 Accuracy of curability expectations

Table 2 depicts accuracy rates by clinical and patient‐reported variables, and Table 3 summarizes patient questionnaire responses. Agreement in accuracy assessments between oncologist reviewers was 100%. Regardless of disease stage, approximately 70% of patients thought it was very or somewhat likely for their treatment to cure their cancer, 80% thought it was very or somewhat likely for their treatment to alleviate cancer‐related problems, and over 90% believed that treatment was very or somewhat likely to prolong their life **(**Table 3 **)**.

**TABLE 2:** Accuracy of curability expectations

	Accuracy rate *N* (%)	Chi‐square *p*‐values
Overall (*n* = 135)	65 (48%)	
Gender		0.29
Males (*n* = 81)	36 (44%)	
Females (*n* = 54)	29 (54%)	
Age		0.29
Younger than 65 years (*n* = 81)	36 (44%)	
65 years or older (*n* = 54)	29 (54%)	
GI cancer type		0.45
Colon (*n* = 36)	22 (61%)	
Pancreatic adenocarcinoma (*n* = 32)	14 (44%)	
Anorectal (*n* = 29)	12 (42%)	
Biliary (*n* = 16)	8 (50%)	
Other (*n* = 22)	9 (41%)	
Disease stage		<0.001
I–III (*n* = 63)	40 (63%)	
IV (*n* = 72)	25 (35%)	
Unresectable disease		<0.001
Resectable (*n* = 57)	38 (67%)	
Unresectable (*n* = 78)	27 (35%)	
Systemic treatment^a^		0.28
Chemotherapy (*n* = 125)	61 (49%)	
Immunotherapy (*n* = 8)	2 (25%)	
Adjuvant chemotherapy for Stage I–III		0.04
No (*n* = 36)	19 (53%)	
Yes (*n* = 27)	21 (78%)	
Visit with surgeon prior to ITU response		0.48
No (*n* = 54)	24 (44%)	
Yes (*n* = 81)	41 (51%)	
DNR status		0.87
No DNR (*n* = 84)	40 (48%)	
DNR (*n* = 51)	25 (49%)	
Information preference^b^		0.14
No detail (*n* = 18)	6 (33%)	
Detail (*n* = 107)	56 (52%)	

This table shows accuracy rates by clinical variables and patient‐reported information preferences. Data presented as *N* (%).

Abbreviations: GI, gastrointestinal; ITU, illness and treatment understanding; DNR, do not resuscitate.

^a^ Two patients did not receive systemic treatment and were excluded. Fisher's exact test results reported due to expected cell counts of <5.

^b^ Unavailable for 10 patients.

**TABLE 3:** Illness and treatment understanding, information preferences, and quality of communication: Questionnaire Patient Responses

Illness and treatment understanding questionnaire				
After talking most recently with your oncologist, how likely do you think it is that your cancer treatment will:	Baseline questionnaire (*N* = 135)		Final questionnaire (*N* = 73)	
Help you live longer?	Very likely	91 (67%)	Very likely	43 (59%)
	Somewhat likely	34 (25%)	Somewhat likely	19 (26%)
	A little likely	5 (4%)	A little likely	9 (12%)
	Not at all likely	2 (1%)	Not at all likely	‐
	No response	3 (3%)	No response	2 (3%)
Cure your cancer?	Very likely	58 (43%)	Very likely	22 (30%)
	Somewhat likely	35 (26%)	Somewhat likely	16 (22%)
	A little likely	12 (9%)	A little likely	9 (12%)
	Not at all likely	1 (1%)	Not at all likely	23 (32%)
	No response^a^	3 (2%)	No response	3 (4%)
Help you with problems you are having because of your cancer?	Very likely	76 (56%)	Very likely	36 (49%)
	Somewhat likely	32 (24%)	Somewhat likely	21 (29%)
	A little likely	8 (6%)	A little likely	5 (7%)
	Not at all likely	7 (5%)	Not at all likely	4 (5%)
	No response	12 (9%)	No response	7 (10%)
**Information and decision‐making questionnaire (*N* = 125)**				
*I like to receive information about my health:*				
(1)^b^	With a lot of detail		107 (86%)	
	As an overview, without much detail		18 (14%)	
(2)^b^	With a family member or friend present		103 (82%)	
	By myself		20 (16%)	
	Both responses selected		2 (2%)	
(3)^c^	By talking with my doctor, nurse, or other healthcare provider		1 (1%)	
	By reading printed information, such as handouts or booklets		48 (48%)	
	By watching a video		60 (61%)	
	By looking for information on the Internet myself		58 (59%)	
	By looking at information on the Internet that was suggested by my healthcare provider		81 (82%)	
**Quality of communication questionnaire (*N* = 60)**				
(1) How often does your care team:				
Listen carefully to you?	Always		58 (97%)	
	Usually		2 (3%)	
	Sometimes		–	
	Never		–	
Explain things in a way you could understand?	Always		53 (88%)	
	Usually		7 (12%)	
	Sometimes		–	
	Never		–	
Give you as much information as you wanted about your cancer treatments?	Always		51 (85%)	
	Usually		8 (13%)	
	Sometimes		1 (2%)	
	Never		–	
Encourage you to ask all the cancer‐related questions you had?	Always		54 (90%)	
	Usually		4 (7%)	
	Sometimes		2 (3%)	
	Never		–	
Treat you with courtesy and respect?	Always		60 (100%)	
	Usually		–	
	Sometimes		–	
	Never		–	
(2) How good is your care team at^*d*^	*Likert Scale Responses: Median (range)*			
Involving you in treatment discussions about your care?	9 (4, 10)			
Asking you about important things in life?	9 (3, 10)			
Asking about spiritual/religious beliefs?	9 (1, 10)			
Discussing your values/goals?	9 (1, 10)			
Discussing whether your care is consistent with your values/goals?	9 (1, 10)			
Talking to you about your feelings concerning the possibility that you might get sicker?	9 (1, 10)			
Talking to you about the details concerning the possibility that you might get sicker?	9 (1, 10)			
Involving you in the decisions about the treatment you want if you get too sick to speak for yourself?	9 (0, 10)			

This table shows responses to illness and treatment understanding, information preferences, and quality of communication questionnaires. Data presented as *N* (%) unless otherwise specified. Empty cells (−) indicate 0 selected responses.

^a^ Three patients who completed the illness and treatment understanding questionnaire did not answer the question regarding curability but were determined to have clearly incurable disease, and these patients were classified as having an inaccurate understanding.

^b^ Instructions indicate to pick one answer only.

^c^ Instructions indicate to pick one or more answers. Twenty‐six patients had no response to question (3) and responses are reported for *n* = 99.

^d^ Responses to these questions were recorded on a 0–10 Likert Scale, along with an option for indicating “did not discuss.” Three patients indicated that they did not discuss any communication aspects related to this question.

Sixty‐five patients (48%) were determined to have accurate expectations for the likelihood of cure from their cancer treatment at baseline (Table 2). Cure accuracy was lower in patients with Stage IV versus Stage I–III disease (35% vs. 63%, *p* < 0.001) and lower in patients with unresectable vs resectable disease (35% vs. 67%, *p* < 0.001). Of Stage I–III patients, accuracy was higher in those who received adjuvant chemotherapy compared to those who did not (21/27, 78% vs. 19/36, 53%, *p* = 0.04). Accuracy did not significantly differ by gender, age, GI cancer diagnosis, type of systemic treatment, or presence of a Do Not Resuscitate (DNR) order. Due to the limited number of variables that demonstrated a statistically significant association with accuracy in the univariable setting, multivariable analysis was not conducted.

Of patients who completed the baseline information preferences questionnaire (*n* = 125), most patients preferred to receive information about their health in detail (86%) and with a family member or friend present (82%) **(**Table 3 **).** The accuracy rate for patients who preferred a general overview was lower than for those who preferred detailed health information (33% vs. 52%, *p* = 0.14); however, these results were not statistically significant **(**Table 2 **).**

Of patients who completed the baseline quality of communication questionnaire (n = 60), nearly all patients selected responses to indicate that they felt heard, were able to share their personal values and goals, had the opportunity to ask cancer‐related questions and discuss their concerns, were provided treatment information in a way that could be understood, and felt included by their care team for decision‐making **(**Table 3 **)**.

### 3.3 Longitudinal evaluation and accuracy differences by end‐of‐life outcomes

Table 4 depicts patterns of understanding over time, showing accuracy and inaccuracy rates at baseline and at time of the final completed questionnaire. Among patients who completed ITU questionnaires at least once after baseline (*n* = 73), 35 (48%) patients had accurate cure expectations at baseline, and this increased to 43 (59%) patients upon completion of the final questionnaire. For the 35 patients with accurate cure expectations at baseline, 28 (80%) remained accurate. For the 38 patients with inaccurate cure expectations at baseline, expectations remained inaccurate for 23 (61%) and were more accurate for 15 (39%).

**TABLE 4:** Longitudinal assessment of curability expectations

	Accuracy at Final ITU questionnaire		
Accuracy at baseline	Inaccurate	Accurate	Total
Inaccurate	23 (61%)	15 (39%)	38 (52%)
Accurate	7 (20%)	28 (80%)	35 (48%)
Total	30 (41%)	43 (59%)	73

This table shows accuracy and inaccuracy rates at baseline compared to time of the final completed questionnaire. Data presented as *N* (%).

Abbreviations: ITU, illness and treatment understanding.

Among patients who died (*n* = 63), baseline accuracy significantly differed by location of death and hospitalization within the last 30 days of life but did not differ if patients received cancer treatment during the last 30 days of life **(**Table 5 **).** Cure accuracy was higher for those who died at home with hospice (56%) compared to those who died in hospitals (14%), or an inpatient hospice facility (20%), or at home without hospice (0%) (*p* = 0.03). Cure accuracy was lower in patients who were hospitalized compared to those who were not hospitalized within the last 30 days of life (25% vs. 59%, *p* = 0.01).

**TABLE 5:** Accuracy rates by end‐of‐life outcomes

	Accuracy rate *N* (%)	Chi‐square *p*‐values
Overall (*n* = 63)	26 (41%)	
Location of death^a^		0.03
Home hospice (*n* = 41)	23 (56%)	
Hospital (*n* = 7)	1 (14%)	
Inpatient hospice (*n* = 5)	1 (20%)	
Home (*n* = 4)	0	
Treatment in the last 30 days of life^b^		0.83
No (*n* = 41)	18 (44%)	
Yes (*n* = 17)	8 (47%)	
Hospitalization in the last 30 days of life^b^		0.01
No (*n* = 34)	20 (59%)	
Yes (*n* = 24)	6 (25%)	

This table shows baseline accuracy rates relative to location of death and care received at the end of life. Data presented as *N* (%).

^a^ Unknown for six patients.

^b^ Unknown for five patients.

## 4 DISCUSSION

In this single‐center, prospective cohort study, we examined the accuracy of curability expectations among patients with GI cancers regardless of stage, and the influence of selected clinical factors on accuracy. Our analysis revealed that half of the patients had accurate baseline expectations. Accuracy of curability expectations was greater in patients with earlier stage disease compared to metastatic disease, in patients with surgically resectable compared to unresectable disease, and in patients with early‐stage disease who received adjuvant chemotherapy compared to those who did not. Accuracy did not differ by type of systemic treatment (immunotherapy vs. chemotherapy), GI cancer diagnosis, or DNR status. Our study contributes real‐time and longitudinal information about illness and treatment expectations from a large cohort of patients, inclusive of all stages of disease, receiving modern‐day cancer therapeutics.

The strengths of our data include disease heterogeneity, high interrater agreement for coding accuracy, and use of questions derived from validated questionnaires.^4^, ^15^, ^17^, ^20^ Moreover, surveys were administered in the setting of our institution's primary palliative care initiative that centered on eliciting patient values as well as assessing for and addressing unmet needs such as uncontrolled symptoms and information about disease and treatment intent.^17^ That is, our assessment of patients' expectations from their cancer treatment did not occur in isolation, but was dovetailed with an endeavor to deliver palliative care and enhance serious‐illness conversations in actual clinical practice, thereby informed by these proactive discussions. As patients had the opportunity to reflect and provide responses longitudinally while receiving their cancer care, our results thus provide up‐to‐date and valuable additions to the evidence on patients' expectations and understanding.

Our methodology was also refined, as each individual patient's disease course and clinical characteristics were reviewed by oncology experts to determine accuracy, instead of categorically classifying survey responses as accurate or inaccurate for patients with advanced disease without likelihood of achieving cure.^15^ For example, when utilizing this more personalized clinical approach, a patient with Stage IV colorectal cancer with a single hepatic metastasis who reported that it was “somewhat likely” that chemotherapy could provide cure would be classified as accurate, whereas this would have been an inaccurate response based on methodology used in Weeks et al.

Our results primarily suggest that patients receiving treatment for metastatic disease may have more difficulty understanding the intent of this treatment than those receiving adjuvant treatment for potentially curable cancer. This may be in part due to existential distress often experienced by patients with metastatic disease, who receive treatment while facing mortality sooner than anticipated or hoped. Yet, it is understandably human for patients to feel this way, especially as drug development continues and as newer therapies reinvigorate optimism for tumor response.^23^, ^24^, ^25^ There are a number of factors that could influence patient perceptions,^16^, ^26^ and prior research has even demonstrated that receiving life expectancy statistics does not impact patients' prognostic expectations.^27^ Additionally, patients with earlier‐stage disease who receive adjuvant chemotherapy may have a better understanding than those who do not, perhaps because oncologists may spend more time to explicitly review the goals of treatment. When oncologists recommend adjuvant chemotherapy for the aim, but not guarantee, of eradicating any residual micro‐metastatic disease, this may lead patients to an accurate understanding of treatment intent more often than in patients in whom no such chemotherapy is prescribed.

Our results also support findings from existing literature on the relationship between information preferences and curability expectations,^28^ the stability of prognostic awareness over time,^29^ and the impact of curability expectations on end‐of‐life care outcomes.^30^ The body of evidence to which the present study adds can help inform how oncology care teams use patients' expectations to guide conversations at times of disease progression and/or clinical decline, with a greater attention on clarifying patients' illness understanding as it relates to their personal values, prognosis, and end‐of‐life care preferences.

A contemporary evaluation of the accuracy of cancer patients' expectations highlights the importance of iterative and tiered (e.g., at diagnosis, recurrence or progression, and near the end of life) communication strategies that support patients as they cope with their illness and prognosis. Even when prognosis is uncertain, oncology care teams should be cognizant of how patients may interpret what has been communicated and how they may perceive their illness, as misconceptions can lead to downstream effects on end‐of‐life care. Ongoing research is needed to develop personalized, simple, and effective methods of conveying prognosis and facilitating understanding, when and as desired by patients and caregivers.^31^, ^32^, ^33^, ^34^ Future communication interventions may require specific roles for nursing professionals,^17^, ^35^, ^36^ oncologists, and palliative care specialists, with a framework that can be acted upon early and that can adapt to all stages and types of cancers.

Our study has several limitations. This study was conducted at a single academic cancer center and our findings may not be generalizable to other healthcare systems. Though MSK's primary palliative care initiative was implemented in only a select number of GI oncology clinics,^17^ we studied a diverse patient population across all stages and GI cancer types. In addition, despite high interrater agreement, a ceiling effect was possible in our methodology; additional reviewers of patient responses may have resulted in a lower agreement level on accuracy assessments. Higher patient‐reported ratings of physician communication quality have previously been associated with less accurate and more unrealistic expectations for the goals of treatment.^15^, ^37^ A ceiling effect may also therefore, explain why most patients reported that their oncology care teams provided high‐quality communication about their health and personhood. While retrospective, end‐of‐life outcomes data were available for almost all study patients. Finally, our analysis focused only on quantitative, Likert‐scale, patient responses to validated surveys on ITU and curability. As described previously,^16^ such analyses may limit the strength of inferences or conclusions, as inaccurate responses may reflect a lack of information, inadequate communication, and/or patient‐level barriers to integration of information or reporting of understanding. Future investigations will incorporate qualitative data and other associations, such as the degree of belief in miracles,^38^ to explain inaccuracies in patient understanding and combine with quantitative responses for mixed‐methods analysis.

In summary, we explored accuracy of curability expectations in patients with GI cancers regardless of stage and identified lower accuracy rates among those with metastatic disease, unresectable disease, and early‐stage patients who did not receive adjuvant chemotherapy. Additional studies will evaluate more disease types and the associations of distress (physical, emotional, spiritual), including worry about death and dying,^39^ on patient expectations. Accurate illness and treatment understanding by patients can support them and their care team to align around the goals and plans for oncologic care.

## AUTHOR CONTRIBUTIONS

Rajiv Agarwal: data curation, formal analysis, writing‐original draft, writing‐review and editing. Paul Shin: conceptualization, data curation, formal analysis, writing – review and editing. Andrea Knezevic: formal analysis, writing – review and editing. Judith E. Nelson: conceptualization, funding acquisition, methodology, supervision, validation, writing‐review and editing. Danielle R. Romano: data curation, investigation, methodology, project administration, writing – review and editing. Camila Bernal: data curation, investigation, methodology, project administration, writing – review and editing. Anjali V. Desai: investigation, methodology, writing – review and editing. Andrew S. Epstein: conceptualization, data curation, formal analysis, funding acquisition, investigation, methodology, supervision, validation, writing‐original draft, writing‐review and editing.

## CONFLICT OF INTEREST

The authors declare no conflicts of interest.

## ETHICS STATEMENT

The Institutional Review Board approved this study and waived informed consent based on minimal risk to participants. The study conforms to recognized standards per the US Federal Policy of Protection of Human Subjects and Good Clinical Practice.

## Data Availability

The data that support the findings of this study are available from the corresponding author upon reasonable request.
